# Just Because (Most) Hospitals Are Publishing Charges Does Not Mean Prices Are More Transparent

**DOI:** 10.2196/14436

**Published:** 2020-02-06

**Authors:** Cody Lendon Mullens, J Andres Hernandez, Evan D Anderson, Lindsay Allen

**Affiliations:** 1 West Virginia University School of Medicine Morgantown, WV United States; 2 Center for Public Health Initiatives University of Pennsylvania Philadelphia, PA United States; 3 The Wharton School University of Pennsylvania Philadelphia, PA United States; 4 Perelman School of Medicine University of Pennsylvania Philadelphia, PA United States; 5 Center for Public Health Initiatives Perelman School of Medicine University of Pennsylvania Philadelphia, PA United States; 6 Department of Health Policy, Management, and Leadership School of Public Health West Virginia University Morgantown, WV United States

**Keywords:** health care costs, delivery of health care, health policy

## Abstract

**Background:**

The Centers for Medicare and Medicaid Services (CMS) recently mandated that all hospitals publish their charge description masters (CDMs) online, in a machine-readable format, by January 1, 2019. In addition, CMS recommended that CDM data be made available in a manner that was consumer friendly and accessible to patients.

**Objective:**

This study aimed to (1) examine all hospitals across the state of Pennsylvania to understand policy compliance and (2) use established metrics to measure accessibility and consumer friendliness of posted CDM data.

**Methods:**

A cross-sectional analysis was conducted to quantify hospital website compliance with the recent CMS policies requiring hospitals to publish their CDM. Data were collected from all Pennsylvania hospital websites. Consumer friendliness was assessed based on searchability, number of website clicks to data, and supplemental educational materials accompanying CDMs such as videos or text.

**Results:**

Most hospitals (189/234, 80.1%) were compliant, but significant variation in data presentation was observed. The mean number of website clicks to the CDM was 3.7 (SD 1.3; range: 1-8). A total of 23.1% of compliant hospitals provided no supplemental educational material with their CDM.

**Conclusions:**

Although disclosure of charges has improved, the data may not be sufficient to meaningfully influence patient decision making.

## Introduction

As of 2017, national health care expenditures in the United States rose to US $3.5 trillion, an increase of close to 4% compared with the previous year [1]. With a stated objective of empowering patients and reducing administrative burdens, the Centers for Medicare and Medicaid Services (CMS) mandated that all hospitals publish charge description master (CDM) data online [2,3]. A hospital CDM is a comprehensive list of a hospital’s charges to patients or health insurance companies for services rendered during a hospital stay. One rationale for the policy is that increased price transparency will encourage patients to *shop around* for competitively priced health care services, much as they would for a new car [4-6].

Recent changes in Medicare’s payment policies under the inpatient prospective payment system (PPS) and the long-term care hospital PPS required that the CDM be made available in a machine-readable format by January 1, 2019 [2]. Only Veterans Affairs (VA) hospitals and hospitals reimbursed under state or local cost control systems are exempt from the requirements. Machine-readable format refers to documents that are digitally accessible and in file formats that are easily processed by computers (ie, comma separated value [CSV] or XML files). Notably, a “frequently asked questions” form published by CMS recommended that CDM data be published in a consumer-friendly, accessible manner, which is an important consideration given that the target consumers of the data are patients [7]. Given the inherent confusion surrounding CDMs—including their relationship to actual prices—even the savviest consumers are unlikely to know how to interpret them [8]. The purpose of this analysis was to assess hospital compliance with the CDM policy and determine accessibility and consumer friendliness of CDM data for all hospitals within the state of Pennsylvania.

## Methods

### Study Design

We examined the presentation of CDM data for all hospitals in the state of Pennsylvania. This state was chosen because of its relatively large number of hospitals and for its variation in rural versus urban health systems, facility types, and hospital tax status. All hospitals were identified using data from the Hospital and Healthsystem Association of Pennsylvania and were categorized as nonprofit, for profit, city, state, or federal based on tax status. In addition, hospitals were classified into the following facility types: general acute, general acute specialty, rehabilitation, psychiatric, VA, long-term acute care, drug/alcohol, maternity, and other specialty hospitals.

### Quantifying Compliance and Consumer Friendliness

Each hospital website was accessed on January 7, 2019, and queried for online CDM publication in a machine-readable format, which was required for compliance with the policy. Hospitals that were noncompliant at initial analysis were reassessed 1 week later on January 14, 2019. Accessibility of the CDMs was determined based on the number of page clicks required to access the CDM from the hospital home page, an established method for assessing website usability and navigability [9,10]. Accessibility of CDMs was further assessed through utilization of hospital website’s search function, using keywords such as “chargemaster,” “charge master,” “charge description master,” “standard charges,” “charge,” and “price.”

To measure consumer friendliness, we next determined if any supplemental information was provided by hospitals to help patients understand the provided materials such as descriptions as to what CDMs are, their utility, or how patients can interpret them in the context of price of care. Finally, all hospital home pages were evaluated based on their display of CDMs or other financial information for viewers and prospective patients. Descriptive statistics were calculated in Stata (StataCorp LLC, version IC 15.1).

## Results

### Hospital Demographics

**Figure 1:**
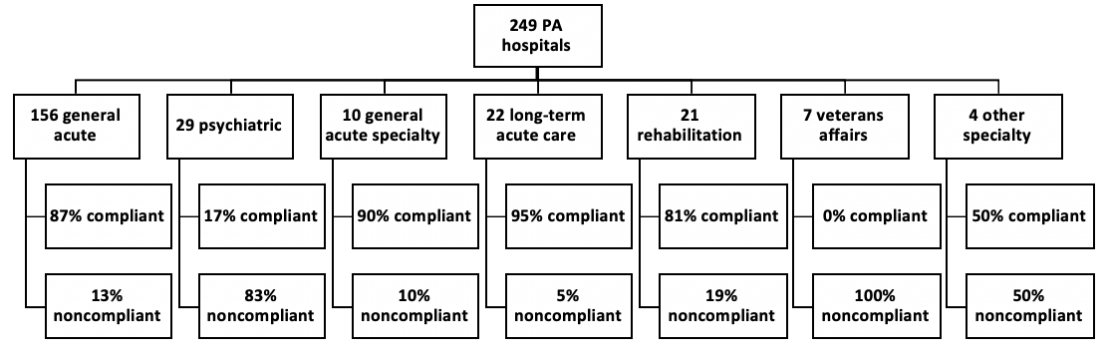
Demographic breakdown of Pennsylvania hospitals and compliance with charge description master policy.

A total of 249 hospitals were identified and included in the study (Figure 1). Most hospitals were nonprofit tax status (148/249, 59.4%), followed by for profit (86/249, 34.5%) and federal/city/state (15/249, 6.1%). The most common hospital type was found to be general acute care hospitals (156/249, 62.7%), followed by psychiatric (29/249, 11.6%), long-term acute care (22/249, 8.8%), rehabilitation (21/249, 8.4%), general acute specialty hospitals (10/249, 4.0%), VA (7/249, 2.8%), and other specialty hospitals (4/249, 1.6%).

### Hospital Compliance With Policy

Most hospitals included CDMs in their website (189/249, 74.9% hospitals). Excluding VA hospitals and hospitals reimbursed under state or local cost control systems, which are excluded from the policy, compliance rose to 80.1% (189/ 234) hospitals. A total of 20 hospitals were out of compliance with the mandated policy because they did not comply with the mandated formatting of CDM data. Moreover, 10 hospitals posted online databases, 1 posted online text, and 9 posted PDFs, none of which were in a machine-readable format as required.

### Analyzing Accessibility and Consumer Friendliness

Among the 189 compliant hospitals, 116 posted billing, pricing, or other financial information (such as payment plans or hospital financial resources) on the hospital home page. Few hospitals included a direct hyperlink or information regarding CDM data and the recent mandate on their hospital website’s home page (21 out of the 189 facilities posting data). Although it was possible to access the CDM through link-clicking on most of the compliant websites, 21 hospitals required users to utilize the search function within the hospital website to obtain their CDM. In addition to the 21 hospitals who only permitted access to CDM data through the search function, an additional 126 hospitals enabled users to query searches within the hospital website to access published CDMs.

**Figure 2:**
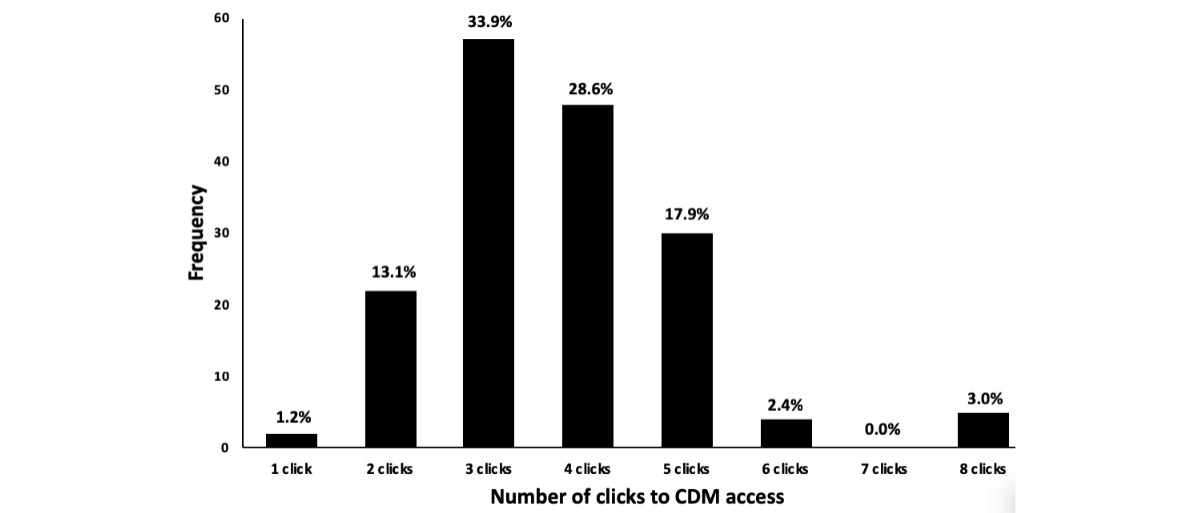
Distribution of hospital website charge description master access based on the number of clicks to download charge description master data. CDM: charge description master.

Mean number of clicks to CDM access was 3.7 (SD 1.3; range: 1-8 clicks; Figure 2). Hospital CDM data end points were predominantly linked and downloadable CSV or XML (files for Microsoft Excel) files (156 hospitals; Table 1).

Finally, we assessed the prevalence of supplemental financial information for users and prospective patients regarding CDMs, cost, charges, and any additional relevant information. Although most hospitals provided supplemental text information on the website for viewers (107/190, ie, 56.3% of compliant hospitals), a substantial proportion (23.1%) did not provide any information. Less commonly, hospitals included text and video information (21/249, 8.4%) or video only (2.1%). Interestingly, 7.9% of hospitals included disclaimers to users or required acknowledgment alluding to the insufficiency of CDMs for determining actual price of care within their text and other supplemental information.

**Table 1:** Hospital website charge description master end points.

Website end point	Hospitals, n (%)
Interactive online database^a^	10 (5.3)
Downloadable comma separated value or XML file	169 (89.4)
Downloadable PDF^a^	9 (4.8)
Online text^a^	1 (0.5)

^a^Interactive online databases, PDFs, and online text are not technically compliant with the stated policy.

## Discussion

### Principal Findings

This is the first known study to quantify compliance with the new CMS CDM transparency requirement. Although 80.1% (189/234) of hospitals in Pennsylvania are compliant with the policy by posting CDM data at the time of our study, wide variation was observed in the degree of accessibility of the CDM and specific compliance with mandated formatting.

One-fifth of Pennsylvania hospitals were noncompliant with the policy, which is not unprecedented; in California, hospitals have been reluctant to comply with regulations mandating that hospitals provide uninsured patients with price estimates to promote health service price shopping [11]. Even with full compliance, however, there are reasons to question the value of the requirements in helping slow the growth of health care costs.

First, consumers of health care struggle to interpret nomenclature around health care financing such as understanding and differentiating between charge and price [12]. A charge is the dollar amount associated with a particular medical service before payer discount negotiation, whereas a price is the negotiated and contracted dollar amount for the payer. In Pennsylvania, many hospitals did not provide accompanying resources with CDM data to help the public understand these differences. Amendments to the policy that require such accompanying resources with CDM data may be helpful and informative to those patients who do access these data.

Second, when consumers are covered by health insurance plans, they are not responsible for negotiating prices of services rendered or bearing the full cost of care. This leads to the dilemma of moral hazard, wherein patients use more medical care than they would had they not been covered by insurance. In the context of health care, providers (ie, hospitals) may feel less obligated to be transparent regarding charges to consumers, realizing that most consumers never bear the full cost of care or the charge associated with CDM. Recent attempts at increasing price transparency to encourage “shopping” in health care have produced mixed results. Sinaiko and Rosenthal [13] studied an insured, nonelderly adult population’s use of a payer-developed payment estimator; they found considerable engagement with and utilization of the estimator, especially among those who were younger, with fewer comorbid conditions, and with relatively high health care system utilization rates. The authors concluded that tools to increase transparency of price in health care have the potential to meaningfully impact patient decision making and health care service utilization [13]. Other studies have failed to find significant changes in actual health care spending associated with implementation and availability of different transparency tools [14,15]. Americans attest to and support the utility of tools for assisting in health care price shopping, but few patients actually seek out health care price–related information in practice [16].

In contrast to consumers who are insulated from much of the costs, many individuals have high deductible health plans, which are consumer-driven in the sense that they bear a much more substantial cost burden when seeking care. This specific population may stand to benefit from published CDM data as a means of shopping for health care services. However, a considerable portion of this population may also be effectively incapable of paying almost any out-of-pocket costs. As a Federal Reserve study recently noted, 40% of Americans do not have the accessible assets to pay a US $400 emergency expense [17]. This underscores both how unpredictable bills can destabilize households and how precarity in household assets can destabilize theories that consumers will vigorously shop for health care services for based on price.

Finally, lack of accessibility and/or consumer friendliness may impede prospective patients from using the CDMs. For example, some CDM data could only be accessed through a focused query using the website’s search function, which patients may not know to use. Other hospitals placed links to the data within unrelated or unlabeled subsections of the website. Moreover, CDM data are generally written in medical jargon likely indecipherable by the general public, using incomprehensive acronyms or technical names of procedures and equipment. This is an important barrier to consider not only for those who have lower health care literacy but also for those who are less adept with computer- and internet-related technologies.

### Future Directions and Limitations

Although the CMS policy is a step in the right direction for increased transparency, the consumer friendliness of these data and the direct implications on patients or their health insurance provider are unclear. One clear benefit of the charge data availability, however, is that researchers will be able to study the significant charge variability that exists between comparable health care facilities and hospitals. Future policies may consider additional steps toward true price transparency for both services rendered as well as pharmaceuticals and medical devices.

Limitations of this study include the single-state analysis; though as mentioned earlier, Pennsylvania was chosen because of its diversity in geography, demographics, and facility types. In addition, because of the cross-sectional study design, it is impossible to make any inferences about the causal relationship between the policy changes and observations about the accessibility of CDM data in Pennsylvania hospitals.

### Conclusions

The majority of hospitals in Pennsylvania have complied with the CDM policy. However, there is considerable variation in the accessibility and consumer friendliness of the CDMs. Determining whether enhanced access to CDM data will alter consumer or institutional behavior remains an important priority for future health services research.

## Abbreviations

CDM: charge description master
CMS: Centers for Medicare and Medicaid Services
CSV: comma separated value
PPS: prospective payment system
VA: Veterans Affairs
